# Wide discrepancy between best practice recommendations and real-life management of suspected eosinophilic esophagitis-associated food bolus impaction

**DOI:** 10.1093/dote/doaf073

**Published:** 2025-09-17

**Authors:** Yangzom Siegfried, Jagoda Pokryszka, Petr Hruz, Simon Buetikofer, Bernhard Morell, Fritz R Murray, Ekaterina Safroneeva, Andrea Kreienbuehl, Thomas Greuter, Alex Straumann, Gerhard Rogler, Luc Biedermann, Alain Schoepfer, Philipp Schreiner

**Affiliations:** Department of Gastroenterology and Hepatology, University Hospital Zurich, Zurich, Switzerland; Division of Gastroenterology and Hepatology, Department of Internal Medicine III, Medical University of Vienna, Vienna, Austria; Department of Gastroenterology and Hepatology, University Center for Gastrointestinal and Liver Diseases St. Clara Hospital and University Hospital of Basel, Basel, Switzerland; Division of Gastroenterology and Hepatology, Luzerner Kantonsspital (LUKS), Lucerne, Switzerland; Department of Gastroenterology & Hepatology, Stadtspital Triemli Zurich, Zurich, Switzerland; Department of Gastroenterology & Hepatology, Stadtspital Triemli Zurich, Zurich, Switzerland; Institute of Social and Preventive Medicine, University of Bern, Bern, Switzerland; Department of Gastroenterology and Hepatology, University Hospital Zurich, Zurich, Switzerland; Division of Gastroenterology and Hepatology, EOC Lugano, Università della Svizzera italiana, Lugano, Switzerland; Department of Gastroenterology and Hepatology, University Hospital Zurich, Zurich, Switzerland; Department of Gastroenterology and Hepatology, University Hospital Zurich, Zurich, Switzerland; Department of Gastroenterology and Hepatology, University Hospital Zurich, Zurich, Switzerland; Division of Gastroenterology and Hepatology, Centre Hospitalier Universitaire Vaudois (CHUV) and University of Lausanne, Lausanne, Switzerland; Department of Gastroenterology and Hepatology, University Hospital Zurich, Zurich, Switzerland; Division of Gastroenterology and Hepatology, Department of Internal Medicine III, Medical University of Vienna, Vienna, Austria

**Keywords:** eosinophilic esophagitis, esophageal, endoscopy, emergency, histopathology, diagnosis

## Abstract

Esophageal food impaction (EFI) is the leading complication in patients with undiagnosed eosinophilic esophagitis (EoE). Limited data exists on pre-hospital care, in-hospital management, and post-hospital follow-up in suspected EoE-associated EFI. This study aims to assess deviations between real-life management and guideline-based recommendations in suspected EoE-associated EFI. This retrospective multicenter study analyzed data from four major Swiss gastroenterology units on patients with EoE-associated EFI. Patients with GERD-related strictures or esophageal cancer were excluded. Data on demographics, emergency department (ED), endoscopy management, and follow-up were obtained from electronic health records. Associations between clinical factors and odds of biopsy were analyzed using logistic regression. Between January 2015 and December 2020, 198 EFI cases (median age 51 years, 29.8% female, 28% with previous EFI) were recorded. Patient delay—the time between symptom onset and ED admission—was ~ 270 minutes. Nearly all patients (94%) required endoscopic bolus removal. The median time from ED presentation to endoscopy was ~150 minutes. Esophageal biopsies were taken in just over half of the individuals (n = 97, 52%), leading to a new EoE diagnosis in 71 (68.9% of those biopsied). Biopsy odds decreased significantly with older age (OR 0.96; 95% CI 0.94–0.98, *P <* 0.05) and known EoE (OR 0.26; 95% 0.09–0.69, *P <* 0.05). Although EoE is a leading cause of EFI, too few patients with a high baseline probability of EoE undergo biopsy in the emergency setting. Among those biopsied, the majority received a new EoE diagnosis, highlighting the importance of histological assessment.

## INTRODUCTION

Eosinophilic esophagitis (EoE) is an immune-mediated disease of the esophagus.^1^ Since the disease is defined not only by symptoms but also by an eosinophilic infiltration of the esophagus, an endoscopy with esophageal biopsy is essential to diagnose EoE.^2^ The cardinal symptom of EoE in adults is dysphagia, with esophageal food impaction (EFI) being one of the main complications and often the reason for first presentation—affecting **~**30% of patients with EoE.^3^^,^^4^ Various studies provide evidence that EoE has become the leading cause of EFI in adults, often requiring emergency endoscopic removal. As the removal process is resource-intensive, the healthcare system is further strained by the rising incidence of EoE and its associated complications, including EFI.^5^^,^^6^ Recent data shows that emergency admissions due to food bolus impactions associated with EoE tripled between 2009 and 2019.^7^

The increasing rates of EoE-related complications, such as EFI, and the subsequent burden on the healthcare system can only be alleviated through proper patient management, beginning with a precise diagnosis. Given that this diagnosis requires histopathological confirmation, performing biopsies during EFI is imperative.^2^ Nevertheless, research has shown that only a minority of patients undergo biopsies at the index endoscopy.^8^^,^^9^ Since EFI and stricture rates increase proportionally with the duration of untreated inflammation in patients with undiagnosed EoE, proper management at disease onset is of utmost importance.^10^ However, diagnostic delay has remained unchanged over the last 30 years, resulting in many patients being at risk for fibrosis and EFI.^11^

Furthermore, recommendations for best practices in the management of EFI differ among endoscopists, and most guidelines lack specific guidance on suspected EoE in cases of EFI.^12^ There is scarce data on in-hospital management and potential factors influencing biopsy taking. Since fear of complications, such as aspiration during EFI removal, is an obstacle to obtaining esophageal biopsies, it can be assumed that patients undergoing elective intubation are biopsied more frequently. Additionally, existing factors such as atopic disease, younger age, and male sex increase the pre-test probability for EoE in EFI cases.^13^

The purposes of this study were to assess the real-world management of EoE-suspected EFIs at four independent tertiary centers in Switzerland and to determine factors influencing the decision to collect biopsies during the emergency procedure.

## METHODS

We performed a multicenter retrospective analysis of consecutive cases of EFI’s suspicious of eosinophilic esophagitis between 01/2015 and 12/2020 at four emergency departments (ED) in Switzerland. We excluded patients with esophageal cancer, known peptic strictures, other non-EoE somatic causes of food impaction, and those with incomplete medical records. The data on demographics, emergency treatment, and follow-ups were obtained from electronic health data systems at each hospital.

Following parameters were documented: age, gender, known EoE, recurrent EFI, year of impaction, atopic disease (y/n), duration of symptoms before ED, therapy before ED, complete dysphagia (swallowing of saliva not possible), type of food as cause for EFI, blood tests (y/n), X-ray (y/n), CT (y/n), ECG (y/n), medication at ED, approximate time to endoscopy (minutes), tracheal intubation with general anesthesia (y/n), endotracheal intubation, retrieval technique, esophageal biopsy (y/n), esophageal dilation, recommended therapy after EFI resolution, histology report (esophageal eosinophilia ≥15 eos y/n), follow-up endoscopy and complications. The diagnosis of EoE was confirmed by the presence of a peak eosinophil count of ≥15 eosinophils/high power field in the esophageal biopsies.

Categorical variables were presented as fractions of total, continuous ones—as medians with interquartile range (IQR). Fisher’s exact test was used to assess the differences in clinical parameters between intubated and non-intubated groups. Association testing was only performed in the group who received endoscopy. To test the primary hypothesis of the impact of intubation on the biopsy number and hospital stay, logistic regression was performed. Due to the retrospective character of the study, no power analysis was conducted. The significance level was set at α = 0.05. No data imputation was performed.

The study was approved by the Ethical Committee of Zurich (No 01158/2021) and conducted in accordance with the Declaration of Helsinki 1964.

## RESULTS

Between January 2015 and December 2020, 331 cases of EFI following admission to an ED were recorded at four Swiss centers. After the exclusion of incomplete cases and ineligible patients (Table 1), data from 198 EFI cases (29.8% female, median age 51 years of age, 28% with previous EFI) were used for further analysis. Fewer than one-fourth (34/198, 17.2%) had a previously established EoE diagnosis. Twelve patients (12/198, 6.1%) did not require endoscopy. EFI characteristics are listed in detail in Table 1.

**Table 1:** EFI characteristics

	Esophageal food impaction (n = 198)
Excluded patients (n = 133)	65.4% (87) incomplete medical record 12.0% (9) cancer 8.0% (6) schatzki ring 8.0% (6) peptic stricture 4.0% (3) Barrett esophagus 1.3% (1) candida esophagitis 1.3% (1) spinal muscular atrophy
Male	70.2% (139)
Age in years (median, IQR)	51 (27.8)
Atopic disease	50.5% (92) (n = 182, 16 patients unknown)
First EFI Second EFI Third EFI Fourth EFI Fifth EFI	75.3% (149) 14.1% (28) 6.1% (12) 3.0% (6) 1.5% (3)
EoE known before EFI	17.2% (34)
Causative Food	75.3% (149) meat 5.1% (10) meat + others 1.5% (3) pill 1.0% (2) bread 1.0% (2) pasta 1.0% (2) potato 1.0% (2) cake 0.5% (1) garlic 0.5% (1) pizza 0.5% (1) lentils 0.5% (1) apple 0.5% (1) granola 0.5% (1) cheese 0.5% (1) cereals

The median time to ED admission was ~270 minutes. Only a minority of patients took any medication before the hospital (Table 2A).

**Table 2:** A. Pre-hospital management

Therapy before admission	12.1% (24) PPI 6.6% (13) STC 1.5% (3) STC and PPI
Time to admission in minutes (median, lq, oq)	270 (122, 660)

**Table 2:** B. Emergency department management

Therapy at emergency department	82.3% (163) none 1.5% (3) PPI 1.0% (2) PPI + corticosteroids 2.0% (4) corticosteroids 1.0% (2) glucagon + others 1.0% (2) Buscopan 1.0% (2) Perfalgan
	0.5% (1) Perfalgan + others 0.5% (1) glucagon 0.5% (1) Temesta
Diagnostics before EFI removal	65.0% (129) blood tests 9.1% (18) barium swallow 4.5% (9) computer tomography 10.1% (20) ECG
First Call	Gastroenterology (71.2%) ENT (22.2%)

In the ED, most patients had blood samples taken, but only a minority had an imaging study before EFI removal (Table 2B).

**Table 2:** C. Endoscopic management

Time to endoscopy in minutes (median, lq, oq)	150 (120, 240)
Elective Intubation	13.6% (27)
Retrieval technique	54.5% (108) push 28.3% (56) pull 1.0% (2) push + pull 9.1% (18) spontaneous gone 0.5% (1) chopped bolus
Devices used	60.6% (120) none 15.7% (31) forceps 5.6% (11) net 1.0% (2) net + snare 2.0% (4) snare 3.5% (7) overtube 3.0% (6) triangle 1.0% (2) cap
Dilation at index endoscopy	1.5% (3)
Biopsy taken	48.9% (97)
Complications	6.1% (12)

Over half of the endoscopists used a push technique (54.5%) to remove the EFI (Table 2C). After the procedure, majority of the endoscopy-reports included a recommendation for a follow-up endoscopy (82.3%). Inpatient admission was necessary in 15.7% (Table 2D), with only 0.62% of admissions being due to iatrogenic mucosal injury of the esophagus during endoscopy or biopsy. The admission rate was not different between patients who received a biopsy versus those who didn’t receive a biopsy (*P* = 0.16).

**Table 2:** D. Post-endoscopy management

New EoE diagnosis	35.8% (71)
Therapy recommended after EFI	19.2% (38) none 53.0% (105) PPI 6.1% (12) PPI + corticosteroids 4.5% (9) PPI + dilatation 4.5% (9) corticosteroids 2.0% (4) PPI + analgesy 1.5% (3) PPI + biopsy 0.5% (1) PPI + antimycotics 0.5% (1) corticosteroids + analgesy
Follow-up endoscopy recommended in report	82.3% (163)
Inpatient admission Reasons for inpatient admission	15.7% (31) 1.6% (5) mucosal lesion due to EFI 1.6% (5) frailty due to age and multimorbidity 0.9 % (3) monitoring due to suspicion of perforation 0.62% (2) oxygen saturation drop 0.62% (2) iatrogenic mucosa lesion (due to overtube) 0.31% (1) respiratory insufficiency 0.31% (1) prolonged waking up period 0.31% (1) already inpatient at the time of EFI 3.41% (11) unknown

**Abbreviations:** EFI, Esophageal Food Impaction; EoE, Eosinophilic Esophagitis; IQR, interquartile range; PPI, proton pump inhibitor; STC, swallowed topical corticosteroids

**Fig. 1:**
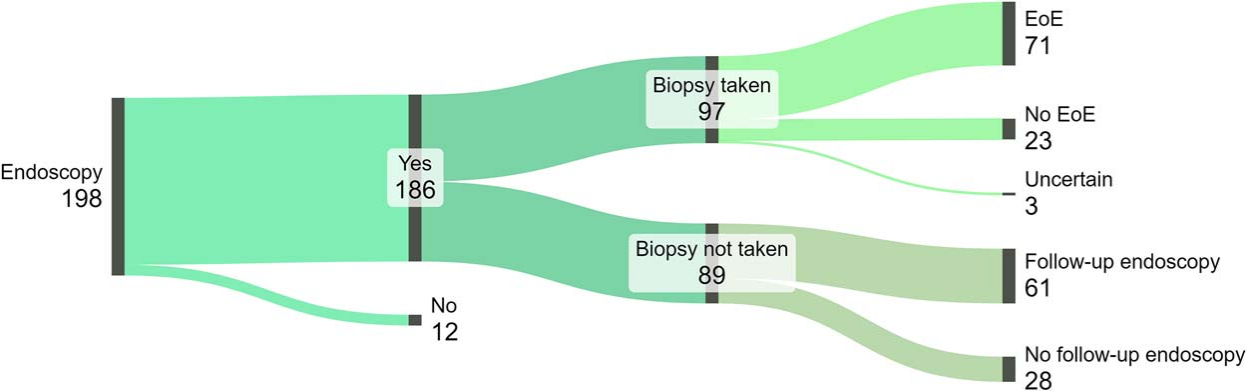
Endoscopy and biopsy in EFI patients. A total of 186 patients with EFI underwent endoscopy. Of these, 97 patients received a biopsy, and 71 of them were diagnosed with EoE as the cause of EFI. Among the 89 patients who did not receive a biopsy during the initial endoscopy, 61 underwent a follow-up endoscopy.

52% of EFI patients who underwent endoscopy received a biopsy during index endoscopy and the majority (68.9%) were diagnosed with EoE as an underlying cause of the food bolus impaction. Among the 48% of patients who did not receive a biopsy during the initial endoscopy, 69% received a follow-up endoscopy (Fig. 1).

In a univariate regression model, there was no association between taking biopsies and general anesthesia with endotracheal intubation, sex, history of atopic disease, and previous EFI. Only increased age and a known diagnosis of EoE were significantly associated with the likelihood of obtaining a biopsy—both showing a negative association (*P* < 0.01, OR 0.96, 95% CI (0.94; 0.98); and *P* < 0.01, OR 0.26 95% CI (0.09–0.69)) (Table 3).

**Table 3:** Obtaining biopsies as outcome

	Univariate analysis			Multivariate analysis		
Variables	OR	95% CI	*P*-value	OR	95% CI	*P*-value
Age (years)	0.96	0.95–0.98	<0.05	0.96	0.94–0.98	<0.05
Male	1.43	0.72–2.88	0.31			
Atopic disease	0.78	0.44–1.40	0.41			
Previous bolus impaction	1.46	0.78–2.77	0.24	2.14	0.87–5.61	0.56
Elective endotracheal intubation	1.66	0.73–3.97	0.24	0.78	0.34–1.76	0.11
Known EoE	0.37	0.16–0.80	<0.05	0.26	0.09–0.69	<0.05

Twenty-seven patients (27/198, 13.6%) received general anesthesia with endotracheal intubation. Neither were they older nor did they have EoE or other atopic diseases more commonly (*P* > 0.05). There were also no differences in sex, previous food impactions, or time to endoscopy. However, the patients who received general anesthesia presented more often with complete dysphagia (*P* = 0.0002).

Admission to the hospital after endoscopy was significantly associated with older age (*P* = 8.6 × 10^−5^, OR 1.05, 95% CI [1.02; 1.07]). This association remained significant after correction for general anesthesia with endotracheal intubation, biopsy taking, symptoms duration and time to endoscopy after ED admission (*P* = 0.02, OR 0.96, 95% CI [0.95; 0.97]) (Table 4).

**Table 4:** Need of hospitalization as outcome

	Univariate analysis			Multivariate analysis		
Variables	OR	95% CI	*P*-value	OR	95% CI	*P*-value
Age (years)	1.05	1.02–1.07	<0.05	1.03	1.01–1.06	<0.05
Symptoms duration before admission (min)	1	0.99–1.00	0.17	1	0.99–1.00	0.06
Time between admission and endoscopy (min)	1	1.00–1.004	0.09	1	1.00–1.01	0.1
Elective endotracheal intubation	2.35	0.89–5.85	0.07	2.18	0.60–7.14	0.21
Obtaining biopsies	0.57	0.26–1.23	0.16	0.57	0.19–1.68	0.32

## DISCUSSION

Parallel with the increasing incidence of EoE, associated complications such as EFI and strictures are also on the rise.^14^ Despite the findings of a recent survey in Europe and the US^12^ about the management and the diagnostic workup of EoE-associated EFI, our study reveals significant gaps in clinical practice. The following findings emerge: (i) In only half of potential EoE-associated EFI cases, indicated biopsies are taken; (ii) Only younger age was significantly associated with increased odds of obtaining biopsies, whereas elective intubation and a high baseline probability of EoE showed no such association; (iii) Biopsy taking is safe and not associated with hospitalization or complications; (iv) The shortcoming in obtaining indicated biopsies cannot be explained by procedure-related risk factors (i.e. non-fasting state of the patient, estimated moderate-to-high aspiration risk), as we did not observe any association with endotracheal intubation before emergency endoscopy; (v) Biopsy rates were significantly lower in patients with a previously established EoE diagnosis. However, in precisely these patients, it is crucial for successful long-term clinical management and prevention of recurrence to identify and address residual or inadequately treated esophageal inflammation, rather than focusing solely on fibrostenosis, reduced distensibility, or a combination thereof; (vi) Only a small percentage of patients who underwent endoscopy were intubated. This contrasts with expert recommendations, which generally advocate for intubation to protect the airway in patients with EFI undergoing endoscopy. It should be emphasized that there is a notable discrepancy in clinical practice between German-speaking countries and the Anglo-Saxon region, where intubation rates, as shown in various studies, are significantly higher.

Our study found that the median time from symptom onset to endoscopy was more than four hours but showed significant variability, likely reflecting differences in symptom severity. Most physicians recommend that if a patient can still swallow saliva, hospital admission does not need to be immediate.^15^ The European Society of Gastrointestinal Endoscopy recommends resolving a subtotal EFI within 24 hours and a complete obstruction within six hours.^16^^,^^17^ However, it must be mentioned that studies conducted according to this recommendation did not demonstrate an association between an increased risk of adverse events and longer times from EFI to endoscopy.^18^^,^^19^ Given that our study found a median delay of over four hours from symptom onset to endoscopy, an important clinical question arises: Is urgent nighttime endoscopy always warranted, or could bolus removal be safely postponed until the following day? Although there appear to be no major safety concerns in most cases nowadays, it must be borne in mind that complete bolus impaction can cause significant discomfort and psychological distress.

Even though most endoscopists do not consider medications, blood tests, or imaging prior to endoscopy to be useful,^12^ in most EFI cases, blood analyses were performed (65.2%). A minority of patients received a diagnostic imaging modality such as computer tomography (CT) scans or an X-ray before EFI removal. It is important to note that none of the mentioned modalities had any therapeutic consequence.

The discrepancy between the recommendations and the clinical practice may be explained by the fact that majority of the patients presenting with EFI in the ED have not been previously diagnosed with EoE. Consequently, ED staff are likely focused on establishing a diagnosis, utilizing laboratory analyses as part of the diagnostic process. However, this practice may ultimately lead to the overutilization of healthcare resources.

The use of medication prior to endoscopy is likely based on the belief that taking some action is preferable to passive observation. However, a randomized controlled study demonstrated that glucagon is not useful in treating EFI^20^ and a small study in patients with suspected EoE-associated EFI showed a resolution rate of only 38% after treatment with budesonide orodispersible tablet.^21^

Since patients in all cases had eaten prior to EFI, the stomach is often not empty during the recommended removal window of six hours. Therefore, most EoE-experienced endoscopists^12^ recommend elective endotracheal intubation to minimize aspiration risk during EFI management. However, endotracheal intubation increases costs and may elevate the risk of complications.^22^ Conversely, other data suggest that these complications may not be directly related to the intubation itself but rather represent a natural consequence of the bolus impaction.^23^ Our study demonstrates a low rate of endotracheal intubation, with no predictive factors associated with its pre-emptive use prior to endoscopy. The current approach appears to favor an individualized evaluation to determine whether intubation is appropriate.^24^ Additionally, intubation was neither associated with obtaining esophageal biopsies nor with hospitalization.

Most guidelines and consensus recommendations now advise taking esophageal biopsies during the index endoscopy in patients with EFI.^12^^,^^25^^,^^26^ However, due to multiple factors—such as endoscopist’s concerns about potential complications, including the risk of perforations, and the limited availability of endoscopy nurses outside regular working hours—recent studies show a low biopsy rate.^9^ Since a significant number of recurrent EFIs are caused by undiagnosed or untreated EoE,^10^^,^^27–29^ failing to identify the underlying cause can result in avoidable healthcare costs—both from repeated emergency endoscopies and the associated emergency staffing. Given that esophageal biopsy remains the only reliable diagnostic tool for detecting EoE, the importance of performing a biopsy at the index endoscopy to establish a diagnosis is undeniable.^30^ Surprisingly, however, our findings do not align with this established data.

We found that in clinical practice, only 52% of EFI patients who underwent endoscopy received a biopsy during index endoscopy. This result is consistent with previous studies showing that only a few endoscopists obtain biopsies during endoscopy.^31–33^ However, among those patients who did receive a biopsy, the majority (77%) were diagnosed with EoE as an underlying cause of EFI.

The only parameters we identified to be significantly—and negatively—associated with the decision to perform a biopsy were a pre-existing EoE and patient age, which is consistent with the findings of another study.^34^ Notably, neither the need for intubation nor the history of previous EFI showed an association with the likelihood of obtaining a biopsy. These findings are counterintuitive, as one might anticipate that intubated patients would provide a better opportunity for performing biopsies and that the urgency of obtaining a diagnosis would be greater in patients with a history of EFI. One potential reason for the low biopsy rates may be a lack of awareness among less experienced endoscopists regarding the importance of histopathological evaluation in diagnosing EoE.

The lower biopsy rate in patients with a pre-existing EoE diagnosis may be attributed to a clinical misconception that EFI is solely a consequence of the pre-existing EoE and that histological evaluation will not impact further management. However, it is essential to differentiate whether the EFI is caused by ongoing inflammation or due to a fibrostenotic complication of EoE. Histological findings can significantly impact subsequent management, potentially leading to an optimization or modification of dietary or medical treatment or necessitating dilation of a fibrostenotic esophagus if no active inflammation is present.

Our study has some limitations. Only patient data from individuals who provided written informed consent for the use of their medical records could be included. However, to minimize selection bias, we analyzed data from multiple hospitals across Switzerland. Furthermore, we were unable to determine the exact number of biopsies taken during endoscopy. Thus, the number of undiagnosed EoE cases is likely even higher, considering that probably not all patients had six biopsies taken.

To sum up, our study provides valuable insights into the current clinical practice of managing EoE-associated EFI. Despite established recommendations emphasizing the importance of early biopsy for accurate diagnosis and effective disease management, we found that a significant number of patients did not receive a biopsy during the index endoscopy. As a result, many patients remained undiagnosed and consequently did not receive the necessary treatment. The lack of timely and appropriate diagnosis not only delays disease control but also increases the risk of future EFIs, further exacerbating the burden on patients and healthcare systems. These findings underscore the need for increased awareness among endoscopists regarding the role of histopathological evaluation in EFI for diagnosing EoE, as well as the importance of adhering to evidence-based guidelines to ensure optimal patient care.

## ABBREVIATION

EoE: Eosinophilic Esophagitis; ED: Emergency Department; EFI: Esophageal Food Impaction; Eos/hpf: Eosinophils per high-power field; Pec/hpf: Peak eosinophil count per high power field.

## Notes

Specific author contributions: Yangzom Siegfried Conceptualization, Methodology, Investigation, Writing – original draft, Writing review & editing. Jagoda Pokryszka Methodology, Investigation, Formal analysis, Writing – review & editing. Petr Hruz Conceptualization, Methodology, Writing – review & editing. Simon Buetikofer Conceptualization, Methodology, Investigation, Writing – review & editing. Bernhard Morell Methodology, Investigation, Writing – review & editing. Fritz R. Murray Methodology, Writing – review & editing. Ekaterina Safroneeva Methodology, Investigation, Writing – review & editing. Andrea Kreienbuehl Conceptualization, Methodology, Investigation, Writing – review & editing. Thomas Greuter Methodology, Investigation, Writing – review & editing. Alex Straumann Conceptualization, Methodology, Investigation, Writing – review & editing. Gerhard Rogler Methodology, Investigation, Writing – review & editing. Alain Schoepfer Methodology, Investigation, Writing – review & editing. Luc Biedermann Methodology, Investigation, Writing – review & editing. Philipp Schreiner Methodology, Investigation, Writing – review & editing.

Financial support: This work was supported by a grant from the Swiss National Science Foundation (32003B_204751/1) awarded to Alain Schoepfer.

Potential competing interests: YS non. FRM none. PS received consulting fees from Abbvie, Falk Pharma, Janssen-Cilag, Takeda, Alfasigma, Pfizer, Sanofi-Aventis, AstraZeneca. E. Safroneeva received consulting fees from Avir Pharma, Inc., Aptalis Pharma, Inc., Celgene Corp., Novartis AG, and Regeneron Pharmaceuticals, Inc. T. Greuter has a consulting contract with Sanofi-Aventis, received a travel grant from Falk Pharma and Vifor, and an unrestricted research grant from Novartis. A.M. Schoepfer received consulting fees and/or speaker fees and/or research grants from Aptalis-Adare-Ellodi Pharmaceuticals, Inc., AstraZeneca AG, Switzerland, Receptor-Celgene-BMS, Dr. Falk Pharma GmbH, Germany, Glaxo Smith Kline AG, Nestlé S.A., Switzerland, Novartis AG, Switzerland, Sanofi-Regeneron Pharmaceuticals, Inc. A. Straumann received consulting fees from Astra-Seneca, BMS-Receptor, Calypso, EsoCap, Falk-Pharma, GSK und Sanofi-Regeneron. L. Biedermann received consulting fees from Vifor, Falk, EsoCap, Pfizer, Sanofi and Calypso and travel fees from Vifor.
